# Human epidermal growth factor receptor 2 expression in gynecologic serous carcinomas and preliminary analysis of its characteristics in patients post-neoadjuvant chemotherapy

**DOI:** 10.3389/fonc.2025.1618237

**Published:** 2026-01-06

**Authors:** Lili Liu, Naixuan Cheng, Fangzhou Kong, Danhua Shen

**Affiliations:** Department of Pathology, Peking University People’s Hospital, Beijing, China

**Keywords:** FISH, HER2, ovarian high-grade serous carcinoma, targeted therapy, uterine serous carcinoma

## Abstract

**Objectives:**

This study aimed to investigate human epidermal growth factor receptor 2 (HER2) protein expression and gene amplification status in uterine serous carcinoma (USC) and ovarian high-grade serous carcinoma (HGSC), determine the frequencies of HER2 overexpression/amplification and HER2-low expression, evaluate two current HER2 interpretation criteria, and analyze the relationship between HER2 status and patient prognosis.

**Methods:**

A total of 51 patients with USC (including eight cases of mixed endometrial adenocarcinoma with predominant serous carcinoma components) and 165 patients with ovarian HGSC (including 43 patients who received neoadjuvant chemotherapy) were retrospectively recruited from the Department of Pathology, Peking University People’s Hospital between January 2019 and January 2025. Clinical and pathological characteristics were summarized. HER2 protein expression in whole-tissue sections was assessed using immunohistochemistry (IHC). HER2 scoring was performed according to two criteria: the 2023 American Society of Clinical Oncology/College of American Pathologists HER2 Testing Guidelines in Breast Cancer and the HER2 Immunohistochemistry Scoring System for Endometrial Serous Carcinoma proposed by the International Society of Gynecological Pathologists (ISGyP). Fluorescence *in situ* hybridization (FISH) was subsequently performed on cases with IHC scores of 1+, 2+, or 3+ for validation. Survival analysis was conducted based on HER2 status.

**Results:**

HER2 gene amplification was detected in 15/51 (29.4%) tissues from patients with USC (including mixed carcinomas with predominant serous morphology). HER2 amplification was identified in 6/122 (4.9%) tissues from patients with primary ovarian HGSC without prior chemotherapy, and in 3/43 (7.0%) tissues from patients with ovarian HGSC with residual disease after neoadjuvant chemotherapy. Evaluation of HER2 IHC results using both interpretation criteria revealed no difference in HER2 3+ expression rates for both USC and ovarian HGSC. However, differences existed in the classification of HER2-low expression (defined as IHC 2+ with negative FISH or IHC 1+). The ISGyP criteria identified more cases with low HER2 expression. For patients with USC, HER2 positivity was not statistically significantly associated with overall survival (OS) or progression-free survival (PFS), although it showed a trend toward higher recurrence. In terms of prognosis, for patients with ovarian HGSC group, HER2 positivity was associated with worse OS (p = 0.049). Among patients with ovarian HGSC after neoadjuvant chemotherapy, HER2 positivity was a strong predictor for shorter PFS (p = 0.003).

**Conclusion:**

A certain proportion of USC and ovarian HGSC cases exhibit HER2 positivity and HER2-low expression. Different interpretation criteria lead to variations in the assessment of HER2 IHC results. The ISGyP criteria can identify more cases with low HER2 expression. Moreover, our findings suggest that HER2 status could be a relevant prognostic marker in these malignancies. Traditional anti-HER2 targeted therapies are indicated for HER2-positive patients, while a broader population of patients with HER2-low expression may benefit from novel anti-HER2 antibody-drug conjugates.

## 1 Introduction

Human epidermal growth factor receptor 2 (HER2) overexpression plays a critical role in the development and progression of breast cancer, gastric cancer, and uterine serous carcinoma (USC) (1–3), and has been established as a standard therapeutic target in breast cancer and a subset of patients with HER2-positive advanced gastric cancer (4, 5).

Gynecologic serous carcinomas primarily occur in the endometrium, ovaries, and fallopian tubes. USC, a rare subtype of endometrial cancer, is highly aggressive and associated with a high recurrence rate, with a 5-year survival rate ranging from 18% to 55% (6, 7). The primary clinical treatments for USC are surgical resection followed by adjuvant chemotherapy. Although USC accounts for only about 10% of endometrial cancers, it contributes to approximately 40% of endometrial cancer-related mortality (7). Conventional treatments, including surgery, radiotherapy, and chemotherapy, have shown limited efficacy. Ovarian high-grade serous carcinoma (HGSC), predominantly originating in the fallopian tubes, is one of the most common and aggressive subtypes of ovarian cancer. Due to its occult location and rapid progression, ovarian HGSC is often diagnosed at an advanced stage, with approximately 95% of patients presenting with metastasis at diagnosis. The 5-year survival rate ranges from 15% to 55% (8). Although some patients achieve remission with chemotherapy, most experience recurrence or metastasis. Therefore, exploring molecular targeted therapies for these tumors has become a major research focus.

HER2 expression in gynecologic tumors, particularly USC, has garnered significant attention. Studies indicate that approximately 29%–35% of patients with USC exhibit HER2 protein overexpression or gene amplification (9). Compared to patients with HER2-negative, patients with HER2-positive have higher recurrence rates and lower survival rates (10), making HER2 an independent prognostic marker for USC. While the anti-HER2 drug trastuzumab alone has not shown significant efficacy in patients with HER2 overexpression or amplification, a phase II trial demonstrated that adding trastuzumab to carboplatin-paclitaxel chemotherapy prolonged progression-free survival in patients with advanced or recurrent HER2-positive USC (11). Similarly, HER2 expression in ovarian HGSC is associated with poor prognosis (12). Therefore, HER2 is expressed in a certain proportion of USC, ovarian HGSC, and recurrent patients who have undergone chemotherapy. Furthermore, novel anti-HER2 drugs, particularly antibody-drug conjugates (ADCs), have shown great therapeutic potential even in tumors with low HER2 expression, largely due to the unique “bystander effect” of ADCs, which allows the drug to kill not only target cells directly bound by the antibody but also penetrate and kill surrounding tumor cells with even lower or negative HER2 expression.

However, accurate clinical assessment of HER2 status faces a significant challenge: the lack of standardized interpretation criteria. In current practice, direct application of the ASCO/CAP guidelines (developed for breast cancer) may yield different results compared to the adoption of the gynecologic-specific ISGyP criteria, particularly when defining the emerging therapeutic subgroup of HER2-low expression.

This study aimed to explore HER2 expression in these two patient groups, compare the evaluation value of the two HER2 interpretation criteria for USC and ovarian HGSC, and assess the predictive value of HER2 status for clinical outcomes.

## 2 Materials and methods

### 2.1 Case selection

This retrospective study enrolled 216 cases, categorized into two groups: 51 patients with USC (including mixed endometrial adenocarcinoma with predominant serous carcinoma components) collected between January 2019 and January 2025, and 165 patients with ovarian HGSC (including 43 patients who underwent neoadjuvant chemotherapy) collected between January 2019 and January 2022, all from the Department of Pathology, Peking University People’s Hospital. Two pathologists, blinded to the fluorescence *in situ* hybridization (FISH) results, reviewed hematoxylin and eosin and immunohistochemistry (IHC) slides to confirm the diagnoses. For the eight cases of mixed endometrial carcinoma with predominant serous components, the “serous” carcinoma areas were evaluated. All cases were staged according to the International Federation of Gynecology and Obstetrics (FIGO) staging system.

### 2.2 IHC

HER2 IHC was performed on whole-tissue sections from all patients using the Ventana PATHWAY anti-HER2 (4B5) platform (Ventana, Beijing, China). HER2 protein expression was scored according to two criteria: the 2023 American Society of Clinical Oncology/College of American Pathologists (ASCO/CAP) HER2 Testing Guidelines in Breast Cancer and the Proposed HER2 Immunohistochemistry Scoring System for Endometrial Serous Carcinoma (ISGyP) (**Table 1**).

**Table 1:** HER2 IHC scoring criteria.

IHC Score	2023 ASCO/CAP	ISGyP
IHC 3+	>10% of tumor cells exhibit strong, complete membrane staining.	>30% of tumor cells exhibit strong lateral, basolateral, or complete membrane staining.
IHC 2+	≤10% of tumor cells exhibit strong, complete membrane staining, or >10% of tumor cells show weak to moderate intensity, complete membrane staining.	≤30% of tumor cells exhibit strong lateral, basolateral, or complete membrane staining, or ≥10% of tumor cells show weak to moderate lateral, basolateral, or complete membrane staining.
IHC 1+	>10% of tumor cells exhibit faint, incomplete membrane staining.	Any proportion of tumor cells with faint, barely visible incomplete membrane staining, or <10% of tumor cells with weak complete membrane staining.
IHC 0	≤10% of tumor cells show faint, incomplete membrane staining, or no membrane staining is observed.	No membrane staining is observed in tumor cells.

According to the ASCO/CAP 2023 guideline, HER2-low expression is defined as tumors with an IHC score of 1+, or an IHC score of 2+ in the absence of HER2 gene amplification.

2023 ASCO/CAP, 2023 American Society of Clinical Oncology/College of American Pathologists (ASCO/CAP) HER2 Testing Guidelines in Breast Cancer; ISGyP, HER2 Immunohistochemistry Scoring System for Endometrial Serous Carcinoma by the International Society of Gynecological Pathologists; IHC, immunohistochemistry.

### 2.3 FISH

HER2 FISH was performed using 4-μm-thick sections from 4% neutral formalin-fixed, paraffin-embedded tissue blocks. The assay was conducted according to the manufacturer’s instructions (Abbott Molecular, Inc., Abbott Park, IL, USA). Tumors were classified as HER2-amplified if the HER2/CEP17 ratio was ≥ 2.0 or the average HER2 copy number per cell was ≥ 6.

### 2.4 Statistical analysis

The prognosis of patients in the HER2-positive, HER2-low, and HER2–0 groups was compared. Progression-free survival (PFS) was defined as the time from surgery to disease recurrence/progression, last follow-up, or death. Overall survival (OS) was defined as the time from surgery to last follow-up or death. The Kaplan-Meier method was used to calculate PFS and OS, and the log-rank test was used to compare survival differences. All statistical analyses were performed using IBM SPSS Statistics 27.0 software.

## 3 Results

### 3.1 HER2 expression status in USC based on ISGyP versus ASCO/CAP criteria

Fifty-one patients with USC (43 pure USC, eight mixed carcinomas with predominant serous morphology) were included. Patient clinical information and tumor characteristics are detailed in **Table 2**. Age ranged from 49 to 88 years (median 66 years). According to FIGO staging, 13 patients (25.5%) had stage I disease, 37 (72.5%) had stage II-IV disease, and 1 (2%) could not be staged. No patients received chemotherapy prior to surgery.

**Table 2:** Summary of patient clinicopathological characteristics.

	USC (n = 51)	Ovarian HGSC (n = 122)	Ovarian HGSC after neoadjuvant chemotherapy (n = 43)
Age range (years) (median)	49–88 (66)	34–79 (58)	43–76 (57)
FIGO stage I	13 (25.5%)	5 (4.1%)	0
FIGO stages II–IV	37 (72.5%), NA (1)†	111 (91.0%), NA (6)†	42 (97.7%), NA (1)†
Tumor size range (cm) (median)	0.8–11.0 (3.7), NA (2)*	0.3–17.5 (7.0), NA (18)*	0–16.0 (4.5), NA (24)*
Presence of vascular invasion	24 (47.1%)	59 (48.4%)	5 (11.6%)
Lymph node metastasis	16 (31.4%), NP (4)**	50 (41.0%), NP (25)**	13(30.2%), NP (13)**
Distant organ metastasis	18 (35.3%), NA (1)‡	106 (86.9%), NA (1)‡	38 (88.4%)

Results are shown as number (percentage) or range (median).

USC, uterine serous carcinoma; Ovarian HGSC, ovarian high-grade serous carcinoma; FIGO stage, International Federation of Gynecology and Obstetrics (FIGO) staging system; NA, not available; NP, not performed.

†Stage was not available.

*Tumor size was not described or otherwise unavailable.

**lymph node dissecting was not performed.

‡Distant metastasis was not available or cannot be determined.

**Figure 1:**
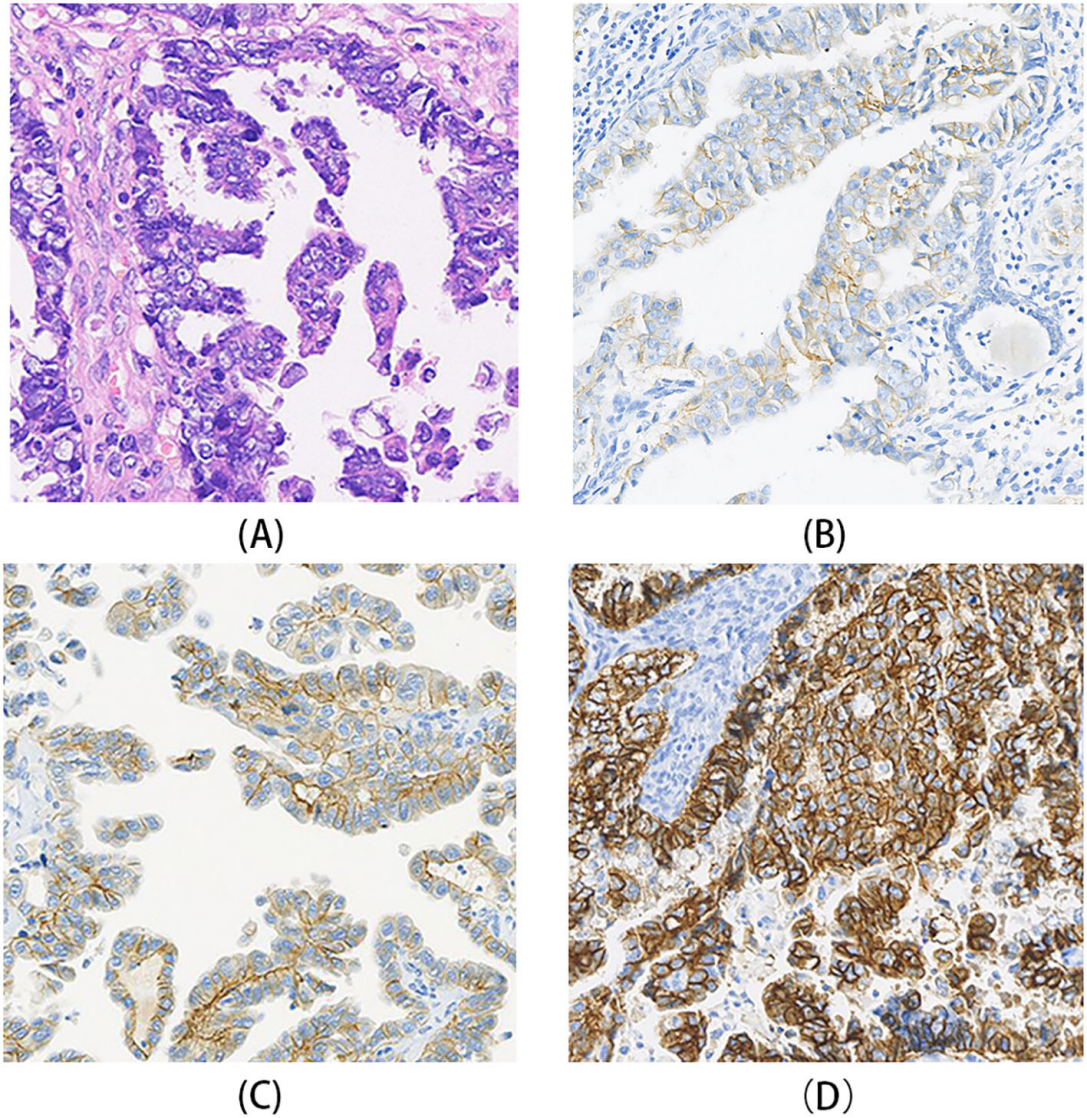
HER2 immunohistochemistry (IHC) staining patterns in gynecologic serous carcinomas. **(A)** A case of USC showing poorly differentiated tumor cells arranged in glandular or solid patterns, with prominent nucleoli. **(B)** HER2 IHC of the case in A, demonstrating weak to moderate basolateral and lateral membrane staining, interpreted as HER2 1+. **(C)** HER2 IHC of another USC case, showing moderate to strong basolateral and lateral membrane staining in most tumor cells, interpreted as HER2 2+. **(D)** A case of ovarian HGSC HER2 IHC of case, exhibiting strong lateral and complete membrane staining, interpreted as HER2 3+.

Using the ISGyP criteria, IHC results were: 8 (15.7%) scored 3+, 22 (43.1%) scored 2+, 12 (23.5%) scored 1+, and 9 (17.6%) were 0. Using the ASCO/CAP criteria, results were: 8 (15.7%) scored 3+, 13 (25.5%) scored 2+, 14 (27.5%) scored 1+, and 16 (31.3%) scored 0 (**Figures 1A-D**). The primary difference was that cases scored as HER2–0 or 1+ by ASCO/CAP criteria were reclassified as 1+ or 2+, respectively, by ISGyP criteria (**Table 3**).

**Table 3:** Comparison of HER2 IHC scoring and expression status using two interpretation criteria.

	USC (n = 51)		Ovarian HGSC (n = 122)		Ovarian HGSC after neoadjuvant chemotherapy (n = 43)	
	A/C	ISGyP	A/C	ISGyP	A/C	ISGyP
IHC 3+	8(15.7%)	8(15.7%)	1 (0.8%)	1 (0.8%)	0	0
IHC 2+	13(25.5%)	22(43.1%)	6 (4.9%)	12 (9.8%)	0	1 (2.3%)
IHC 1+	14(27.5%)	12(23.5%)	16 (13.1%)	28 (23.0%)	3 (7.0%)	9 (20.9%)
IHC 0	16(31.3%)	9(17.6%)	99 (81.1%)	81 (66.4%)	40 (93.0%)	33 (76.7%)
HER2-positive	15(29.4%)	15(29.4%)	6(4.9%)	6(4.9%)	3(7.0%)	3(7.0%)
HER2-0	15(29.4%)	9(17.6%)	98(80.3%)	81(66.4%)	38(88.4%)	33(76.7%)
HER2-low	21(41.2%)	27(52.9%)	18(14.8%)	35(28.7%)	2(4.7%)	7(16.3%)

USC, uterine serous carcinoma; Ovarian HGSC, ovarian high-grade serous carcinoma; A/C, 2023 American Society of Clinical Oncology/College of American Pathologists (ASCO/CAP) HER2 Testing Guidelines in Breast Cancer; ISGyP, HER2 Immunohistochemistry Scoring System for Endometrial Serous Carcinoma by the International Society of Gynecological Pathologists; IHC, immunohistochemistry.

**Figure 2:**
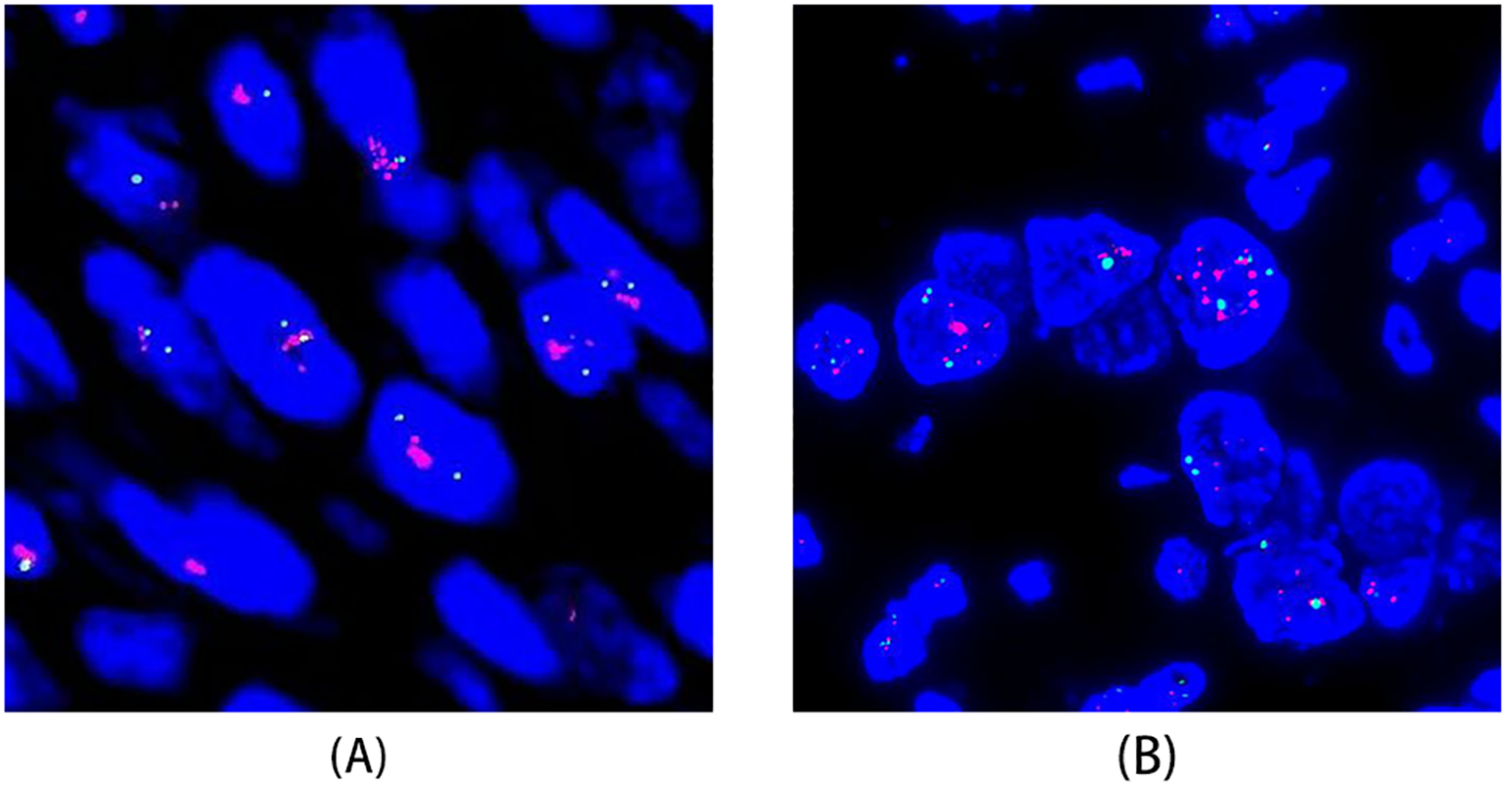
HER2 gene amplification assessed by fluorescence in situ hybridization (FISH). **(A)** FISH of a USC case showing HER2 gene amplification. **(B)** FISH of an ovarian HGSC case demonstrating significant heterogeneity in HER2 expression, with focal areas of HER2 gene amplification.

FISH analysis was conducted on 42 cases scored as HER2 1+ or higher according to the ISGyP criteria. HER2 gene amplification was identified in 15 cases (29.4%)(**Table 3**, **Figure 2A**). Among these FISH-positive cases, seven exhibited IHC HER2 3+, four were IHC HER2 2+, one were IHC HER2 1+, and one case was classified as HER2–0 by ASCO/CAP criteria but as HER2 1+ by ISGyP criteria. Additionally, two cases were categorized as HER2 2+ under the ISGyP criteria but as HER2 1+ using the ASCO/CAP criteria. Of the 22 USC cases interpreted as IHC 2+ by ISGyP criteria, six were confirmed to be FISH-positive. This amplification frequency is consistent with previously reported data. Based on the ISGyP criteria, the prevalence of HER2-low expression (defined as IHC 1+ or IHC 2+ without gene amplification) was 52.9%. In contrast, using the ASCO/CAP criteria, the HER2-low expression rate was 41.2%. This discrepancy primarily stems from the reclassification of a subset of ASCO/CAP HER2–0 cases as HER2 1+ under the ISGyP guidelines(**Table 3**).

### 3.2 HER2 expression status in ovarian HGSC based on ISGyP versus ASCO/CAP criteria

#### 3.2.1 Primary ovarian HGSC (without prior chemotherapy)

This study included 122 patients with primary ovarian HGSC. Patient clinical information and tumor characteristics are detailed in **Table 2**. Age ranged from 34 to 79 years (median 58 years). According to FIGO staging, most patients were in advanced stages: 5 (4.1%) had stage I disease, 111 (91.0%) had stage II-IV disease, and 6 (4.9%) could not be staged. No patients had received chemotherapy prior to surgery.

Using the ASCO/CAP criteria, IHC results were: 1 (0.8%) scored 3+, 6 (4.9%) scored 2+, 16 (13.1%) scored 1+, and 99 (81.1%) were 0. Using the ISGyP criteria, results were: 1 (0.8%) scored 3+, 12 (9.8%) scored 2+, 28 (23.0%) scored 1+, and 81 (66.4%) were 0 (**Table 3**). The HER2 3+ interpretation was consistent between criteria.

FISH was performed on 41 cases scored as HER2 1+ or higher by ISGyP criteria. HER2 amplification was detected in six cases (4.9%): one IHC 3+ case, two IHC 2+ cases, one IHC 1+ case, one case scored as HER2 1+ by ASCO/CAP but HER2 2+ by ISGyP, and one case scored as HER2–0 by ASCO/CAP but HER2 1+ by ISGyP. Among the 12 HGSC (without prior chemotherapy) cases scored as IHC 2+ by ISGyP criteria, three were confirmed FISH-positive. Based on the ISGyP criteria, the prevalence of HER2-low expression was 28.7%. In contrast, using the ASCO/CAP criteria, the HER2-low expression rate was 14.8%. This finding indicates that the ISGyP criteria can identify additional cases with potential gene amplification among those classified as HER2-low or HER2–0 by the ASCO/CAP criteria, which is consistent with the conclusion drawn from the uterine serous carcinoma cohort (**Table 3**).

Notably, significant heterogeneity in HER2 expression was observed in ovarian HGSC. In the HER2 3+ case, strong membrane staining (3+) was present in only about 7% of the tumor area, which corresponded to the FISH-amplified region, while most other areas scored 0 (**Figure 2B**).

#### 3.2.2 Ovarian HGSC after neoadjuvant chemotherapy

Forty-three patients with ovarian HGSC who had residual disease after neoadjuvant chemotherapy were included. Patient clinical information and tumor characteristics are detailed in **Table 2**. Age ranged from 43 to 76 years (median 57 years). All patients were FIGO stage II–IV.

Using the ASCO/CAP criteria, all 43 cases were HER2–0 or 1+: 40 (93.0%) were HER2 0, and 3 (7.0%) were HER2 1 +. Using the ISGyP criteria, 1 (2.3%) was scored 2+, 9 (20.9%) were scored 1+, and 33 (76.7%) were 0. No cases were scored 3+ by either criterion. FISH was performed on 10 cases scored as HER2 1+/2+ by ISGyP criteria, revealing HER2 amplification in three cases (7.0%): one case scored as HER2 1+ by both criteria, and two cases scored as HER2–0 by ASCO/CAP but HER2 1+ by ISGyP. Based on the ISGyP criteria, the prevalence of HER2-low expression was 16.3%. Using the ASCO/CAP criteria, the HER2-low expression rate was 4.7% (**Table 3**).

### 3.3 Correlation between HER2 status and clinical prognosis

For the prognostic analysis, the ISGyP scoring system was applied to define HER2 status, as it recognizes disease-specific staining patterns and offers superior sensitivity for detecting HER2-low expression. This ensured a consistent and biologically relevant stratification. Patients were accordingly classified as HER2-positive, HER2-low, or HER2–0 to assess the association between HER2 expression levels and clinical outcomes.

**Figure 3:**
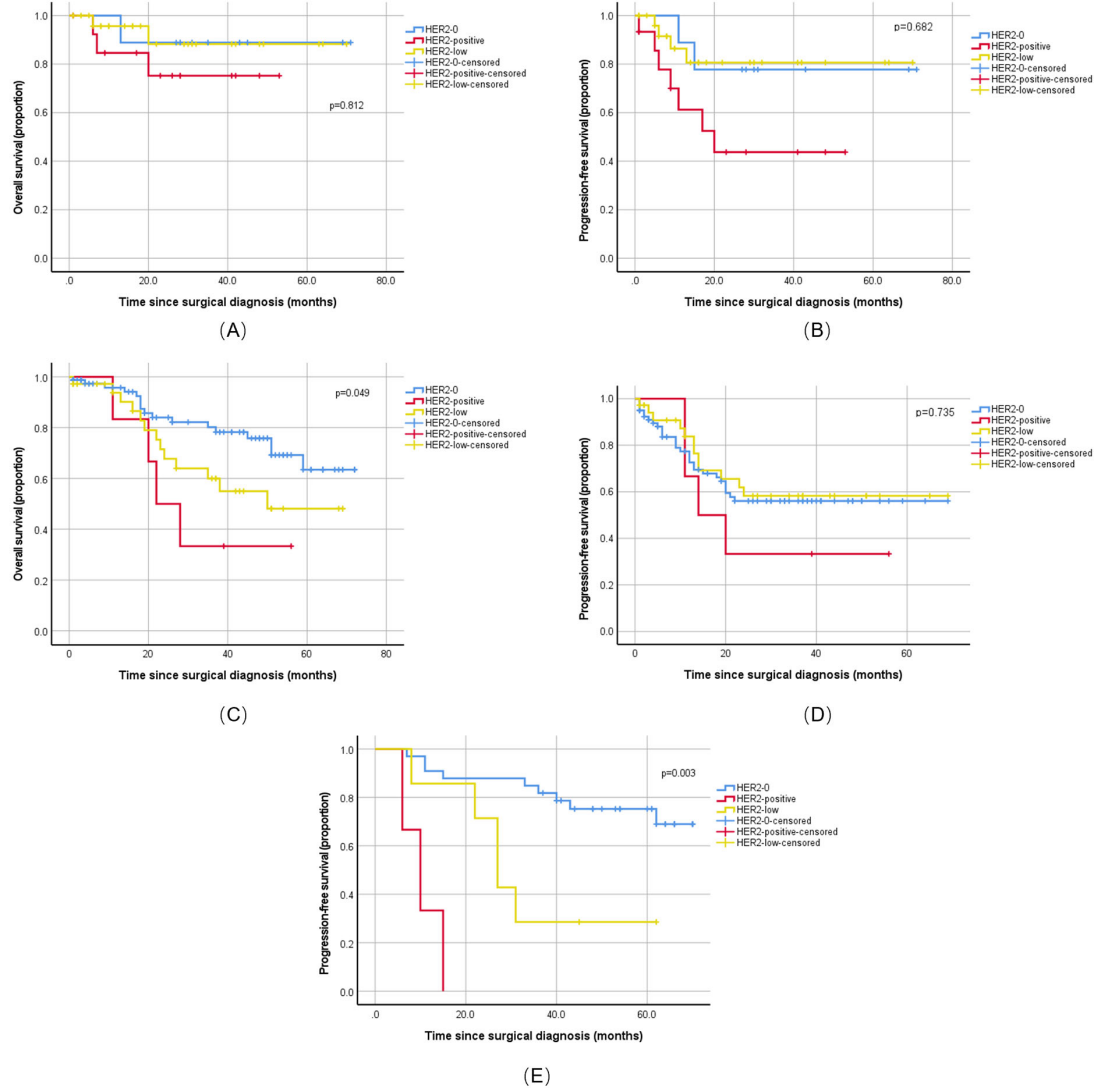
Prognostic impact of HER2 status in ovarian and uterine serous carcinoma. Kaplan-Meier survival curves comparing progression-free survival (PFS) and overall survival (OS) among patients with HER2-positive, HER2-low, and HER2-0 status. **(A)** OS in the uterine serous carcinoma cohort (n=51). No significant difference was observed (Log-rank p=0.812). **(B)** PFS in the uterine serous carcinoma cohort (n=51). No significant difference was observed (Log-rank p=0.682). **(C)** OS in the primary untreated ovarian high-grade serous carcinoma (HGSC) cohort (n=122). A significant difference was observed (Log-rank p=0.049). **(D)** PFS in the primary untreated ovarian HGSC cohort (n=122). No significant difference was observed (Log-rank p=0.735). **(E)** PFS in the ovarian HGSC cohort following neoadjuvant chemotherapy (n=43). A significant difference was observed (Log-rank p=0.003). P values were determined by the Log-rank test. HER2-0, HER2-low, and HER2-positive groups are represented by solid, dashed, and dotted lines, respectively.

Among the 51 patients with USC, HER2 status showed no statistically significant association with 2-year OS or PFS (OS: p = 0.812; PFS: p = 0.682). However, the HER2-positive group (n = 15) showed worse disease control, with a disease progression rate of 46.7% (7/15), much higher than other subgroups, and a median PFS of 20.0 months (**Figures 3A, B**), numerically the shortest among all groups, suggesting that HER2 positivity might be a potential risk factor for disease progression.

Among the 122 patients with primary ovarian HGSC, HER2 status was an independent prognostic factor for 5-year OS (log-rank p = 0.049). Patients who are HER2-positive (n = 6) had a poor prognosis, with a median OS of only 22.0 months, significantly shorter than the HER2–0 and HER2-low groups. Regarding PFS, although the difference was not statistically significant, this group also showed the worst trend, with the numerically shortest 2-year median PFS and a high disease progression event rate of 66.7% (4/6) (**Figure 3C**–**D**), further indicating that HER2 positivity is not only a negative indicator for long-term survival but also closely related to the risk of early recurrence after treatment.

Among the 43 patients with ovarian HGSC who received neoadjuvant chemotherapy, HER2 status was a strong predictor for PFS (log-rank p = 0.003). Although the sample size was small, all patients in the HER2-positive group (n = 3) experienced disease progression, with a median PFS of only 10.0 months (**Figure 3E**), strongly suggesting that it is a key risk factor for early recurrence after chemotherapy. However, due to the low number of OS events, the current data could not assess the impact of HER2 status on OS.

## 4 Discussion

With in-depth research on the role of HER2 in the development and progression of solid tumors, anti-HER2 therapy has become a new option for advanced stages of these malignancies, demonstrating good efficacy. Recent studies show that HER2 is expressed in gynecologic tumors, and the latest research indicates that HER2 is associated with poor prognosis for both OS and PFS (13), especially in USC. Our data analysis showed that although HER2 positivity in USC was not statistically significantly correlated with OS or PFS, potentially due to limited sample size and statistical power, it was associated with poor disease control. Therefore, HER2 should be routinely tested in USC as a therapeutic target. A phase II clinical trial by Fader et al. (11) showed that patients with advanced or recurrent USC treated with trastuzumab plus chemotherapy had significantly improved PFS and OS compared to chemotherapy alone, particularly for stage III–IV patients. Consequently, the anti-HER2 drug trastuzumab has been formally recommended as a treatment option for USC.

Reported rates of HER2 protein overexpression in USC range from 14% to 80%, and gene amplification rates from 21% to 47% (1, 14–22), primarily due to limitations in tissue sample selection and differences in interpretation criteria. Using breast or gastric cancer HER2 interpretation criteria to assess HER2 expression in USC has led to significantly varied results. Buza et al. (23) compared two cutoff values (10% and 30%) for defining IHC 3+ (HER2-positive) status; the 30% cutoff showed higher concordance with FISH results and greater inter-pathologist agreement. In our cases, using both the breast cancer and ISGyP criteria, the HER2 3+ positivity rate was 15.7%. The small sample size might explain why differences in identifying HER2 3+ positive cases between the two criteria were not evident in this study.

HER2 protein expression in USC exhibits unique characteristics, often manifesting as basolateral or lateral membrane staining, particularly in areas with glandular differentiation. This staining pattern is more commonly seen in gastric adenocarcinoma and micropapillary breast carcinoma (24–26). When applying breast cancer HER2 interpretation criteria to USC, it is crucial to consider this unique expression pattern. Cases with strong basolateral/lateral membrane staining in ≥10% of tumor areas can be interpreted as 3 +. In our cases, basolateral and lateral membrane staining patterns were interpreted as equivalent to complete membrane staining in breast cancer. Cases classified as HER2 3+ using the 10% cutoff were also classified as 3+ using the 30% cutoff. This indicates that for identifying HER2 protein-positive (IHC 3+) cases in USC, both the breast cancer and ISGyP criteria are applicable, provided the unique staining pattern is recognized. However, for interpreting scores from 0 to 2+, the ISGyP criteria are more inclusive of the unique expression patterns in USC and provide broader criteria for 1+ and 2+ interpretations. In our cohort, the HER2-low expression rate was 41.2% using ASCO/CAP criteria and 52.9% using ISGyP criteria. This suggests the ISGyP criteria can identify a higher proportion of cases with low HER2 expression. A similar trend was observed for ovarian HGSC: HER2-low rates were 14.8% (ASCO/CAP) vs 28.7% (ISGyP).

Lu et al. (27) retrospectively analyzed the impact of adding trastuzumab to chemotherapy on OS in patients with advanced HER2-positive USC. Their results showed that adding trastuzumab to first-line carboplatin/paclitaxel chemotherapy significantly improved OS. For gynecologic malignancies, especially USC, the necessity of screening for HER2-positive cases is widely recognized. The HER2 positivity rate in our USC cohort was 29.4%, consistent with previous studies (19). However, because this is a retrospective study, clinical efficacy data for targeted therapy are not yet available. Literature reports that HER2 overexpression in ovarian cancer is associated with significantly decreased overall survival (28). Our results show that patients with HER2-positive ovarian HGSC have a worse prognosis, with significantly shorter OS and PFS compared to HER2–0 and HER2-low groups, demonstrating that patients with ovarian HGSC can benefit from anti-HER2 therapies, particularly novel anti-HER2 ADCs (29). Reported rates of HER2 protein overexpression or gene amplification in ovarian HGSC range from 2–4% (30), while our study found a HER2 gene amplification rate of 4.9% in primary ovarian HGSC, slightly higher than previous reports, possibly because we included one IHC 3+ case with significant intratumoral heterogeneity, although its positive area was <30% of the cutoff. Based on the DESTINY-PanTumor02 phase II trial (31), trastuzumab deruxtecan showed encouraging antitumor activity in patients with HER2 IHC 1+ or 2+ endometrial and ovarian cancers. Reported rates of HER2-low expression in USC are inconsistent, approximately 30%–50% (31, 32), and in ovarian cancer are around 17%–24% (32, 33). We believe the main reasons are the lack of unified interpretation criteria and significant intratumoral heterogeneity. Given the refinement of HER2-low interpretation guidelines in breast cancer, we hope that standardized criteria more suitable for the expression characteristics of gynecologic tumors, particularly for HER2-low interpretation, will be established soon.

The DESTINY-PanTumor02 phase II trial found that ADC drugs can also prolong OS in heavily pretreated patients with ovarian cancer, with a median OS of 13.2 months for all patients. This offers new hope for patients with limited treatment options, refractory or recurrent disease. During our collection of ovarian HGSC samples, we noted that due to anatomical features and the occult nature of ovarian HGSC onset, some patients present with metastasis at diagnosis and cannot undergo primary surgery, requiring neoadjuvant chemotherapy followed by interval surgery. Our follow-up data showed that HER2 positivity was significantly associated with PFS (P = 0.003), with a median PFS of only 10.0 months. Although our sample size was small, all three patients with HER2-positive in this subgroup experienced disease progression. Therefore, we believe that for these patients, who often develop resistance to first-line platinum/taxane chemotherapy and have limited options, finding new effective therapeutic targets is most urgent. Therefore, we included this group to explore HER2 positivity or HER2-low expression, providing preliminary data for future large-scale clinical trials. We acknowledge that including these patients might cause confusion, and we clarify that this is an exploratory but clinically meaningful part of our study. We believe future research on HER2 expression in post-treatment recurrent patients will be even more significant.

Our study has some limitations. First, our study is limited by its single-center retrospective design, additionally, the use of archived tissue sections for IHC and FISH analysis has inherent limitations. Future multi-center prospective studies are needed to overcome these limitations. Second, due to the relative rarity of USC, our single-center data might be subject to bias. Third, as novel anti-HER2 ADC drugs have not yet been approved for use at our center, we lack efficacy comparison data before and after ADC treatment. However, we believe that more comprehensive future clinical trials will continue to validate the therapeutic potential of ADCs for HER2-low gynecologic malignancies, especially for refractory and recurrent patients, offering them new hope.

In conclusion, our data show that both USC and ovarian HGSC have varying proportions of HER2-positive and HER2-low cases. For traditional HER2-targeted therapies, patients who are HER2-positive are the primary candidates. For novel ADCs, HER2-low expression can provide effective treatment for a broader patient population, particularly for those with recurrent or advanced refractory disease after prior therapy. Therefore, standardizing HER2 testing and interpretation criteria for endometrial and ovarian cancers is extremely important.

## Data Availability

The original contributions presented in the study are included in the article/Supplementary Material. Further inquiries can be directed to the corresponding author.
